# Applying Resin Radial Injection for Manufacturing Fiber-Reinforced Polymer Composite: Advanced Mathematical Modeling and Simulation

**DOI:** 10.3390/polym16243525

**Published:** 2024-12-18

**Authors:** Joel S. Oliveira, Laura H. Carvalho, João M. P. Q. Delgado, Antonio G. B. Lima, Antonildo S. Pereira, Célia M. R. Franco, Francisco S. Chaves

**Affiliations:** 1 Department of Materials Engineering, Federal University of Campina Grande, Campina Grande 58429-900, PB, Brazil; joel.silva@estudante.ufcg.edu.br (J.S.O.); heckerdecarvalho@yahoo.com.br (L.H.C.); 2 CONSTRUCT-LFC, Department of Civil Engineering, Faculty of Engineering, University of Porto, 4200-465 Porto, Portugal; 3 Department of Mechanical Engineering, Federal University of Campina Grande, Campina Grande 58429-900, PB, Brazil; antonio.gilson@ufcg.edu.br (A.G.B.L.); samuelchaves28@gmail.com (F.S.C.); 4 Federal Institute of Education, Science and Technology of the Sertão Pernambucano, Salgueiro 56000-000, PE, Brazil; antonildo.pereira@ifsertao-pe.edu.br; 5 Department of Physics and Mathematics, Federal University of Campina Grande, Cuité 58175-000, PB, Brazil; celia.maria@professor.ufcg.edu.br

**Keywords:** fluid flow, porous media, composite, RTM, analytical and numerical simulation

## Abstract

Recently, the liquid composite molding technique (LCM) has been used for producing fiber-reinforced polymer composites, since it allows the molding of complex parts, presenting good surface finishing and control of the mechanical properties of the product at the end of the process. Studies in this area have been focused on resin transfer molding (RTM), specifically on the resin rectilinear infiltration through the porous preform inserted in the closed cavity neglecting the sorption effect of the polymeric fluid by the reinforcement. Thus, the objective of this work is to predict resin radial flow in porous media (fibrous preform), including the effect of resin sorption by fibers considering a one-dimensional approach. For correct prediction of the flow behavior inside the porous media, an advanced modeling approach composed of the mass conservation equation and Darcy’s law is used, and the solution of the coupled equation is obtained. Transient results of the flow front location, velocity and pressure within the mold during the resin infiltration are shown, the effects of different parameters for resin (viscosity), reinforcement (sorption term, permeability and porosity) and process (injection pressure and injection radius) are analyzed, and an in-depth discussion is performed.

## 1. Introduction

Composites are materials obtained by combining two or more different materials, one of which is a reinforcement (filamentary, fibrous or particulate), which is dispersed and embedded in a matrix in different types of arrangement [1,2,3]. Mechanical performance of the composite is related to the reinforcement/matrix interfacial area available for stress transfer and interaction between the involved phases. For example, the mixture of reinforcement and matrix will modify different properties such as stiffness, mechanical resistance, and thermal properties, and provide protection against degradation [4].

Currently, high-performance and low-weight materials, including polymer composite reinforced by fiber, are being used in the automotive, sports, construction, and other industries [5]. However, there are various methods for manufacturing polymer matrix composites, and RTM (resin transfer molding) is one of them. In this manufacturing technique, a thermosetting resin (liquid phase) is injected into a dry fibrous reinforcement inserted into a closed mold until the mold is completely filled. This is followed by the resin curing process and demolding. Details of this procedure can be found easily in the cited references [6,7,8,9].

During the injection process, the resin flows through a reinforcement and impregnates the fibers. To analyze this class of physical problem, the current literature reports different analytical and numerical methods and laboratory experimentation. Through experiments, it is possible to analyze real process parameters, but it can be expensive or impossible to carry out. On the other hand, analytical methods have serious limitations, as they can only be applied to simple geometries and especially to problems whose simplification of hypotheses does not deviate too much from the results of the real physical phenomenon. However, both are crucial for validation of most advanced techniques such as numerical methods (computational fluid dynamics).

Due to the high level of complexity of the fibrous media and physical characteristics of the resin, mathematical modeling of the flow of resin in a porous medium is not an easy task. The modeling involves the mass conservation equation and Darcy’s law under different initial and boundary conditions. Despite limitations, the exact solution of the mathematical model equations has been frequently used to predict the fill time, poor impregnation points and the best resin injection and exit points quickly and to estimate the resin mobility and porous media permeability.

For years, there have been several books [10,11,12,13], book chapters [14,15,16] and articles [17,18,19,20,21] devoted to composite manufacturing using RTM techniques through experiments and simulations and applied to different mold shapes.

In their book, Rudd et al. [10] related the potential benefits of the liquid composite molding process, including RTM, the different variants of the RTM technique and important information about the process technology, such as resin flow behavior and cure under the experimental and theoretical aspects, and process control.

In their book chapter, Advani and Hsiao [15] commented about the role of the liquid composite molding process emphasizing different aspects such as the physics of the process and modeling (fluid flow, heat transfer and cure processes) and simulation as tools for process optimization and flow control.

Keller and co-authors [18] noticed the behavior of fast-cure epoxy systems. Research was conducted to identify the processing constraints that different variations of the compression resin transfer molding process present when using fast-curing resins. In this research, the authors developed a robust multiphysics model to compare three process variations—gap injection, direct dosing and wet pressing—studying their fluid flow and exothermic reaction. The proposed modeling showed the importance of the injection strategy to avoid excess temperature during the injection process.

Despite the efforts of many researchers related to the progress in flow modeling in the RTM process, there are visible gaps in the published studies. For example, the vast majority of the 1D simulation studies have been carried out in rectilinear infiltration, and few studies are related to radial infiltration, particularly when considering the effect of resin sorption by the reinforcement. Furthermore, due to the complex preforms’ architecture and pore-size structure, in real-world situations, the resin flow occurs at the macroscopic and microscopic levels. Thus, flow modeling at the microscopic level considerably increases the level of mathematical formulation, solution of the governing equations and flow phenomena analysis.

In addition to the information already explained, Parnas and Phelan [22] and Sadiq et al. [23] reported the important role of evaluating the interactions between outside flow and inner flow in the fiber during resin absorption by fibrous media. In their studies, they verified some trends, which reinforces the importance of new studies on this issue. Thus, many mathematical models to predict resin flow in porous preforms have been developed, highlighting how the global flow behavior is affected by the resin infiltration in the fiber [24,25,26,27,28,29,30,31,32,33].

Resin sinking during the impregnation of porous preforms is not an imaginary phenomenon, but a true reality. Experimental observations in transparent mold allow easy visual verification of the resin flow behavior along the fiber preform. For example, images of the fiberglass preform without the resin have shown that it is originally light in color, and as resin infiltration progresses, it slowly becomes darker. This suggests that the saturation of the fiber by the resin is not instantaneous, but this phenomenon occurs gradually [34].

Based on the information cited in the text, the aim of this research is to theoretically study the flow of resin in fibrous media fully filled by air, taking into account the sorption term of the resin by the fibers. This requires the mass conservation equation (that includes sink term) coupled with an appropriate Darcy´s law equation. Here, applying a radial infiltration procedure, the RTM technique was used considering a one-dimensional approach. The innovation in this work is related to the inclusion of the sink term incorporated in the mass conservation equation, making it possible to verify the effect of this parameter on the pressure history and other hydrodynamic parameters of the radial injection process. We believe that the simplified mathematical model proposed here is very useful in assisting academics and engineers in making decisions about manufacturing composites by applying the RTM technique.

## 2. Materials and Methods

### 2.1. The Physical Problem and the Geometry

**Figure 1:**
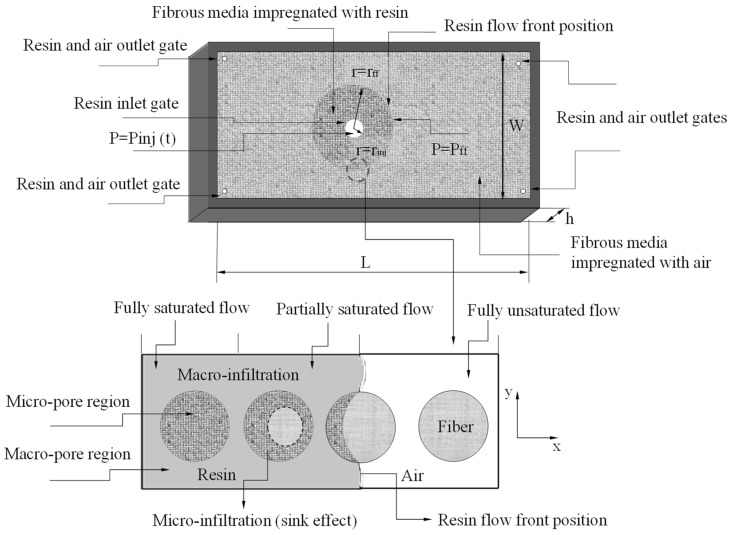
Schematic configuration of resin radial flow within the fibrous preforms, highlighting the fully saturated, partially unsaturated, and fully unsaturated regions of resin and the sink effect.

This study deals with the radial flow of resin through a rigid fibrous porous medium (reinforcement) placed in a closed mold cavity, as illustrated in Figure 1. Initially, all void spaces inside the mold (macrovoids) and fibrous media (microvoid) are fully filled by air. In radial infiltration, the resin is injected through an inlet port of not null radius r_inj_ located at the center of the mold, and it flows through the fibrous reinforcement until it touches the wall of the mold. From this point, the resin flows in a two-dimensional way, until leaving the mold by ventilation points placed close to the border.

During the transient injection process, resin flowing through the fibers tends to force air to flow out of the mold cavity. As the resin passes over the fiber, the pressure of the resin outside the fiber forces it into the fiber.

### 2.2. The Model

For physical problems involving single-fluid flow including sink term, the mass conservation equation is given as follows [12,13]:

(1) $$\frac{\partial \rho }{\partial t}+\nabla \cdot \left(\rho \vec{v}\right)=-\bar{S},$$

If the fluid is considered to be Newtonian and incompressible with constant density and viscosity, Equation (1) can be rewritten assuming the following form:

(2) $$\nabla \cdot \vec{v}=-\frac{\bar{S}}{\rho },$$

Now, if radial injection and the term of resin sorption by the fibers are considered, Equation (2) can be rewritten in cylindrical coordinates as follows:

(3) $$\frac{1}{r}\frac{\partial }{\partial r}\left(r\rho v_{r}\right)=\bar{S},$$

In Equations (1)–(3), ρ represents the density, v_r_ represents the velocity, and $\bar{S}$ corresponds to the sink term (mass flow rate of resin absorbed by the reinforcement).

Since the fluid is considered incompressible (constant density), Equation (1) can be written as follows:

(4) $$\frac{1}{r}\frac{\partial }{\partial r}\left(rv_{r}\right)=-\frac{\bar{S}}{\rho },$$

Due to the low resin velocity and the small pore size often found in preforms during resin injection (RTM process), inertial effects in the momentum conservation equation may be negligible. Thus, the Darcy–Brinkman’s equation can be used to model Newtonian fluid flowing in porous media [35,36,37,38,39,40]. Furthermore, because the small viscous shear effects of the resin during fluid flow through the porous structure and the small value of the porous media permeability, Darcy–Brinkman’s model can be reduced to the well-known Darcy´s law. This way, fluid velocity through porous media will be represented only by terms involving pressure gradient and gravitational effects (Darcy’s law) as reported in Equation (5):

(5) $$\vec{v}=-\frac{k}{\mu }(\nabla p-\rho \vec{g}),$$

where $\vec{g}$ represent the gravity acceleration vector, k corresponds to porous media permeability, and μ is the viscosity. In Equation (5), the pressure corresponds to the sum of the static pressure and the capillary pressure.

The permeability of porous media can be explained physically as a measure of the ease with which a single-phase fluid moves in a porous medium under the effect of a dynamic pressure gradient, including gravitational effects. When two or more immiscible phases flow within a porous medium, great interaction occurs at the interfaces between the fluids, causing different velocities between the phases and capillary effects (capillary pressure), thus modifying the local pressure and fluid saturation. Then, the pressure drop produces an apparent change in permeability throughout the porous medium.

The permeability of a porous medium can be determined in laboratory by experiments using Darcy’s law under steady-state conditions (or through estimation using empirically derived formulas) by forcing a known fluid to flow within a specified porous medium under the action of a pressure gradient.

Considering capillary and gravitational effects to be negligible, Darcy’s law takes the following form:

(6) $$\vec{v}=-\frac{k}{\mu }\nabla p,$$

For radial flow, Equation (6) can be written as follows:

(7) $$v_{r}=-\frac{k}{\mu }\frac{\partial P}{\partial r},$$

Note that the pressure gradient has a very large value at the beginning of the infiltration process, because dr has a very small value. With the progress of resin injection process, pressure gradient decreases, and therefore, the flow front velocity decreases in time.

Following this, replacing Equation (7) into Equation (4), the following is obtained:

(8) $$\frac{1}{r}\frac{d}{dr}\left(\frac{k}{\mu }r\frac{dP}{dr}\right)=\frac{\bar{S}}{\rho },$$

or

(9) $$\frac{1}{r}\frac{d}{dr}\left(\frac{k}{\mu }r\frac{dP}{dr}\right)=s,$$

where $s=\frac{\bar{S}}{\rho }$ represents the sink term due to delayed saturation of fibrous media compared to the empty spaces (pores) between the surrounding fibers. The term $\bar{S}$ represents the rate of local resin infiltration per volume unit from macrovoids in the fibrous preform to microvoids in the fiber.

Equation (8) can be rewritten as follows:

(10) $$\frac{d}{dr}\left(\frac{k}{\mu }r\frac{dP}{dr}\right)=rs,$$

By integrating both sides of Equation (9) twice with respect to r, considering permeability and viscosity as constant, we obtain the following result:

(11) $$P=s\frac{\mu }{k}\frac{r^{2}}{4}+C_{1}\frac{\mu }{k}\ln r+C_{2},$$

The integration constant C_1_ and C_2_, are obtained considering the following boundary conditions:

(a) P = P_inj_ at the injection port.

(b) P = P_ff_ at the resin front position.

Thus, using the boundary conditions into Equation (11), and after some mathematical procedure, we obtain

(12) $$P=\frac{s\mu }{4k}\left(r^{2}-{r_{ff}}^{2}\right)+\left\{\left[P_{inj}\left(t\right)-P_{ff}\right]+\frac{s\mu }{4k}\left({{{r_{ff}}^{2}-r}_{inj}}^{2}\right)\right\}\frac{\ln\left(\frac{r}{r_{ff}}\right)}{\ln\left(\frac{r_{inj}}{r_{ff}}\right)}+P_{ff},$$

Using Equation (12), it becomes possible to obtain the transient pressure field in the circular region completely filled by resin. On the other hand, from the derivative of Equation (12), one obtains the following:

(13) $$\frac{dP}{dr}=\frac{s\mu }{2k}r+\left\{\frac{\left[P_{inj}\left(t\right)-P_{ff}\right]+\frac{s\mu }{4k}\left({{{r_{ff}}^{2}-r}_{inj}}^{2}\right)}{\ln\left(\frac{r_{inj}}{r_{ff}}\right)}\right\}\frac{1}{r},$$

Then, substituting Equation (13) into Equation (7), the resin mean velocity can be determined by the following:

(14) $$v_{r}=-\frac{k}{\mu }\left\{\frac{s\mu }{2k}r+\left[\frac{\left(P_{inj}\left(t\right)-P_{ff}\right)+\frac{s\mu }{4k}\left({{{r_{ff}}^{2}-r}_{inj}}^{2}\right)}{\ln\left(\frac{r_{inj}}{r_{ff}}\right)}\right]\frac{1}{r}\right\},$$

The resin’s real velocity (interstitial velocity) through the porous media (macrovoid) is given by the following:

(15) $$v_{real}=\frac{V_{r}}{\varepsilon }=\frac{-k}{\varepsilon \mu }\left\{\frac{s\mu }{2k}r+\left[\frac{\left[P_{inj}\left(t\right)-P_{ff}\right]+\frac{s\mu }{4k}\left({{{r_{ff}}^{2}-r}_{inj}}^{2}\right)}{\ln\left(\frac{r_{inj}}{r_{ff}}\right)}\right]\frac{1}{r}\right\},$$

where ε is the porous media porosity (macroporosity). Fiber porosity (microporosity) is implicitly included in the sink term. For instance, $\varepsilon =1-V_{f}$, V_f_ being the fiber volume fraction.

Using Equation (15), the volumetric flow rate of resin during the injection process will be given by the following:

(16) $$Q_{inj}=v_{real}\times 2\pi r_{inj}h,$$

where h corresponds to the mold cavity thickness.

According to Figure 1, the resin flow front location at any time can be calculated considering that in the location where r = r_ff_, the resin velocity will be $v_{real}=\frac{dr}{dt}$. Thus, Equation (15) can be rewritten as follows:

(17) $$\frac{dr}{dt}=\frac{-k}{\varepsilon \mu }\left\{\frac{s\mu }{2k}r+\left[\frac{\left[P_{inj}\left(t\right)-P_{ff}\right]+\frac{s\mu }{4k}\left({{{r_{ff}}^{2}-r}_{inj}}^{2}\right)}{\ln\left(\frac{r_{inj}}{r}\right)}\right]\frac{1}{r}\right\},$$

or

(18) $$r(t)\frac{dr(t)}{dt}=\left[-\frac{s}{2\varepsilon }\right]{r(t)}^{2}+\left\{\frac{k}{\varepsilon \mu }\left[P_{inj}\left(t\right)-P_{ff}\right]-\frac{s}{4\varepsilon }r_{inj}^{2}\right\}\frac{1}{\ln\left(\frac{r(t)}{r_{inj}}\right)}+\left[\frac{s}{4\varepsilon }\right]\frac{{r(t)}^{2}}{ln\left(\frac{r(t)}{r_{inj}}\right)},$$

For s = 0, it is possible to obtain the exact solution of Equation (18). On the other hand, if s ≠ 0, the exact solution of Equation (18) is not possible and the use of numerical solution becomes necessary. Herein, to obtain the numerical solution of Equation (18), we use the software Mathematica^®^, Version 10.

### 2.3. Geometrical and Thermo-Physical Parameters

To keep the radial flow in progress, two conditions can be used: the injection pressure or injection velocity (volumetric flow rate). In this research only, we applied the first condition. Table 1 presents all geometric parameters of the mold and thermo-physical parameters of the fibrous medium and resin and also summarizes the simulation data used for all cases analyzed.

## 3. Results and Discussions

### 3.1. Validation

#### 3.1.1. Theoretical Case

Validation of the results presented in this work can be easily verified by making the sink term null, i.e., s = 0, in Equations (12), (14) and (18). Using this procedure, the following results can be easily obtained:

(a) Pressure field

(19) $$P(t)=\left[P_{inj}\left(t\right)-P_{ff}\right]\frac{\ln\left(\frac{r}{r_{ff}}\right)}{\ln\left(\frac{r_{inj}}{r_{ff}}\right)}+P_{ff},$$

The results presented in Equation (19) are according to the published literature [10].

(b) Velocity field

(20) $$v_{r}(t)=-\frac{k}{r\mu }\left[\frac{\left(P_{inj}\left(t\right)-P_{ff}\right)}{\ln\left(\frac{r_{inj}}{r_{ff}}\right)}\right],$$

The results presented in Equation (20) are according to with the published literature [41].

(c) Flow front position

(21) $$r(t)\frac{dr(t)}{dt}=\frac{k}{\varepsilon \mu }\frac{\left[P_{inj}\left(t\right)-P_{ff}\right]}{ln\left(\frac{r(t)}{r_{inj}}\right)},$$

From Equation (21), by considering a radial flow under constant injection pressure (P_inj_ = constant) and separating the variables, and integrating both sides of the obtained equation at the interval t = 0 (where r = r_inj_ and P = P_inj_) to t = t_ff_ (where r = r_ff_ and P = P_ff_), the following is obtained:

(22) $$t_{ff}=\frac{\mu \varepsilon }{2k\left(P_{inj}-P_{ff}\right)}\left[r_{ff}^{2}ln\left(\frac{r_{ff}}{r_{inj}}\right)-\frac{1}{2}\left(r_{ff}^{2}-r_{inj}^{2}\right)\right],$$

in accordance with the reported literature [10,12]. In Equation (22), t_ff_ represents the filling time corresponding to the resin flow front location r_ff_, as already specified in Figure 1.

#### 3.1.2. Experimental Case

Luz [9] carried out some experiments on soybean oil radial impregnation in a fiber preform (E-glass fiber) in a closed mold (300 × 300 × 20 mm^3^). The fluid density and dynamic viscosity are 914 kg/m^3^ and 58.6 cP (23 °C). The mold contains one injection gate and four outlet gates.

The resin’s injection pressure in the mold was monitored during each experiment. The transient pressure behavior is given by Equation (23) obtained by fitting to the experimental data:

(23) $$P_{inj}\left(t\right)=\left\{\begin{array}{l} P_{0}+a_{1}t^{0.25}+a_{2}\exp\left(\frac{a_{3}t}{a_{4}+a_{5}t}\right),\;\mathrm{for}\;0\leq t\leq t_{e}\\ P_{e},\;\mathrm{for}\;t>t_{e} \end{array}\right.$$

where P_0_ = 1013.23 mbar is the atmospheric pressure. The parameters that appear in Equation (10) are represented in Table 2.

**Figure 2:**
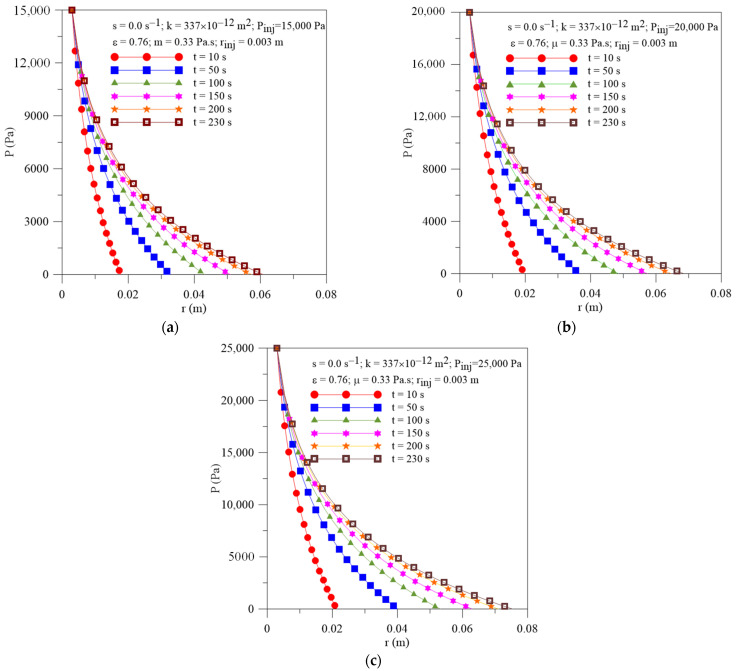
Transient behavior of pressure inside the mold for different resin injection pressure. (**a**) Case 01, (**b**) Case 02 and (**c**) Case 03.

During the transient infiltration process, the radius of the flow front was measured. The following data were obtained in the experiments: porosity = 0.583 and t_border_ = 560 s. Figure 2 illustrates the volume fraction distribution at the moment where the resin touches the first wall of the mold (t_border_).

On the other hand, since injection pressure was given as a function of time in Equation (23), we determined the average value for injection pressure along the entire process until t = 560 s, obtaining a constant value 1704.79 mbar (170.479 kPa) for this parameter, which was used in the simulation.

Table 3 presents a comparative between experimental t_border_ and the predicted value, showing the respective relative percentual error in relation to the experimental data and the values of the porous media permeability and the sorption term obtained in the simulation after fitting to experimental data of the fluid flow front position.

Comparing the values of t_border_, it can be verified that there are no errors superior to 0.1%, proving the robustness of the proposed modeling to predict radial flow behavior. Furthermore, comparing the predicted values of the porous media permeability considering the sorption term to be null [9] or not null (this work), we can verify 26% deviation between the predicted values. It is possible to notice the low value found for the sorption term according to impermeable fiber reinforcement used in the experiment (E-glass fiber).

### 3.2. Hydrodynamic Analysis of the Studied Cases

#### 3.2.1. Effect of Process Parameters

Figure 2a–c show the radial behavior of the pressure within the preform at different moments of the resin impregnation process and P_inj_ = 15, 20 and 25 kPa, respectively, considering the resin sink term to be null. By analyzing this figure, a transient non-linear behavior of this parameter is observed, for all studied cases, as reported in the literature [34,42,43,44,45], but different from the linear behavior presented by resin pressure history in rectilinear infiltration [10]. Furthermore, the largest pressure gradients inside the mold can be easily observed at the beginning of the process, being progressively reduced with increasing time and radial position. These phenomena occur due to the large difference between the resin pressure at the injection gate and the pressure at the exit gate during the impregnation process, as well as due to the amount of resin inside the mold, which increases with processing time. In these figures, the point where the curve touches the horizontal axis corresponds to the position of the resin flow front (r_ff_) at each moment of the injection process.

**Figure 3:**
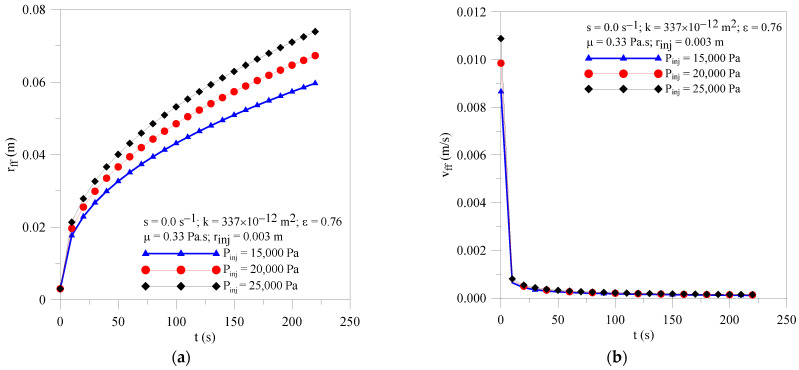
Transient behavior of (**a**) resin flow front location and (**b**) resin velocity for the Cases 01, 02 and 03.

In Figure 3a is illustrated the transient history of the resin front radial location for injection pressure of 15, 20 and 25 kPa. By analyzing Figure 3a, it is verified that, for fixed moment, the higher the pressure injection, the higher the radial location of resin front from the injection point, proving the role of this hydrodynamic parameter in the resin impregnation process. Furthermore, as can be seen, there is a large difference in values of this parameter for the three flows studied herein. The difference between results increased over time. It is important to mention that the intensity of this effect is related to, but not limited to, the parameters of the porous medium (mobility, tortuosity, permeability and porosity) and the resin (viscosity).

Pressure gradients strongly affect the resin velocity field inside the preform. Figure 3b illustrates the transient history of the resin front velocity for different values of the injection pressure. From the analysis of this figure, an asymptotic behavior of the resin velocity can be seen, decreasing with the process time and tending to constant value for long process times. This behavior can be verified for all studied cases. In general, the higher the injection pressure, the higher the velocity of the resin inside the mold, especially at the beginning of the injection process; however, the influence of this parameter decreases strongly for long times.

**Figure 4:**
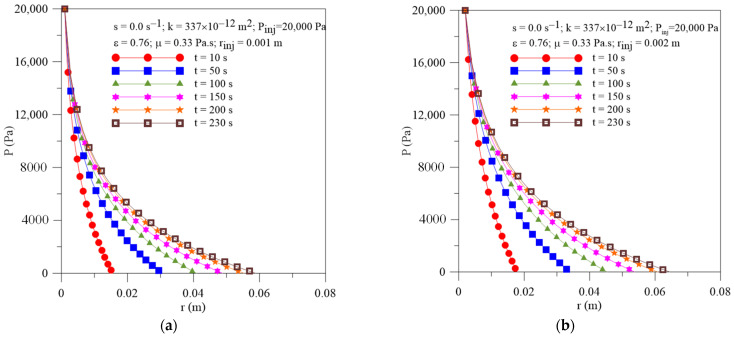
Transient behavior of pressure inside the mold for different resin injection radii. (**a**) Case 04 and (**b**) Case 05.

Figure 4a,b displays results of the radial behavior of pressure within the preform at different moments of the injection process and injection radius. The results show similar behavior to previous results already shown in Figure 2. As expected, a transient and non-linear behavior of this parameter along the process is verified for all studied cases. Furthermore, we can see the higher values of the pressure gradients within the mold cavity at the beginning of the resin impregnation process.

In these figures, the point where the curve touches the horizontal axis corresponds to the position of the resin flow front (rff) at each moment of the injection process.

**Figure 5:**
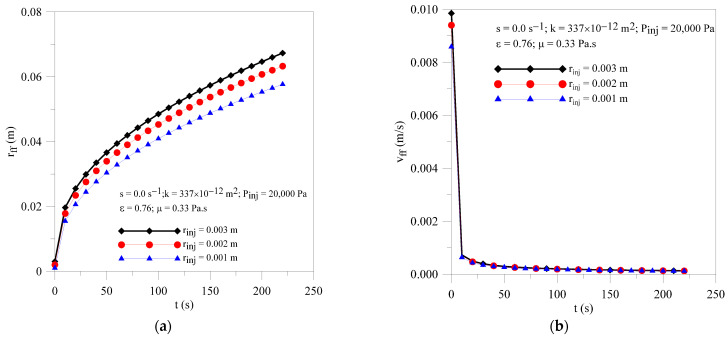
Transient behavior of (**a**) resin flow front position and (**b**) resin velocity for the Cases 02, 04 and 05.

In Figure 5a is illustrated the transient behavior of the resin front radial location for injection pressures of 15, 20 and 25 kPa. Analyzing Figure 5a, it can be seen that the greater the injection radius, the greater the radial location of the front of the resin from the injection port, due to the increase in the mass flow rate of injected resin. It is important to mention that the intensity of this effect is related to, but not limited to, the parameters of the porous medium (mobility, tortuosity, permeability and porosity) and the resin (viscosity). Pressure gradients strongly affect both the resin front radial location and resin velocity field inside the preform, especially at the initial moments of the process. However, the influence of the injection radius in the resin velocity inside the preform is strongly reduced for long injection times.

#### 3.2.2. Effect of Fibrous Medium Parameters

**Figure 6:**
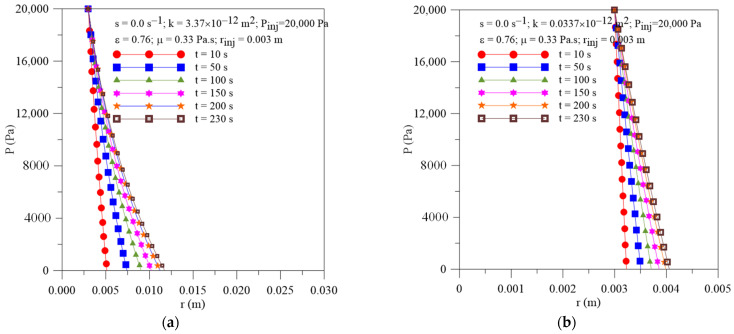
Transient behavior of pressure inside the mold for different porous medium permeabilities. (**a**) Case 06 and (**b**) Case 07.

Figure 6a,b show the radial behavior of the pressure within the preform at different moments of the resin impregnation process and k = 3.37 × 10^−12^ and 0.0337 × 10^−12^ m^2^, respectively, considering resin sink term to be null. From an analysis of this figure, a transient and non-linear behavior of this parameter is verified along the process, for all studied cases. At the beginning of the process, greater pressure gradients are observed inside the mold cavity, decreasing with increasing radial position. From the comparison between Figure 2 and Figure 4, we affirm that the permeability parameter of the porous medium has a greater influence on the local pressure values than the injection pressure and the injection radius.

**Figure 7:**
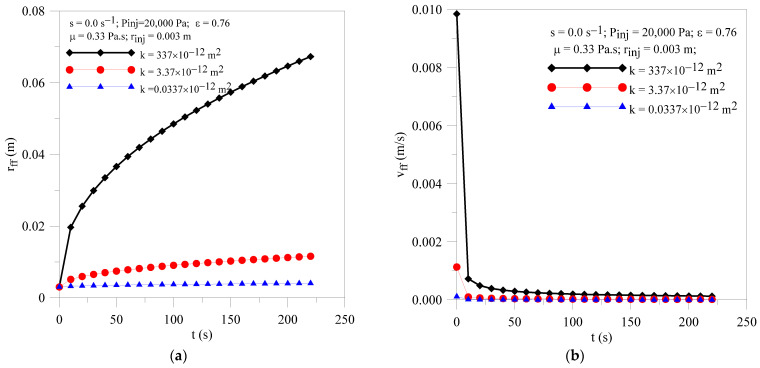
Transient behavior of (**a**) resin flow front position and (**b**) resin velocity for the Cases 02, 06 and 07.

Figure 7a shows the transient behavior of the resin flow front radial position for different values of porous medium permeability (k = 337 × 10^−12^, 3.37 × 10^−12^ and 0.0337 × 10^−12^ m^2^). When analyzing this figure, it is observed that, once the injection moment is fixed, the greater the permeability value of the porous medium, the further away from the injection point the resin flow front will be. From the comparison between Figure 3 and Figure 5, we affirm that the permeability parameter of the porous medium has a greater influence on the location of the resin front than the injection pressure and injection radius.

Figure 7b highlights the transient behavior of the resin flow front radial velocity for different values of the porous medium permeability. From the analysis of this figure, an asymptotic decrease in resin velocity with injection time can be observed, tending to a constant value for long process times. It can be seen that the lower the permeability of the porous medium, the lower the resin velocity values, due to the greater difficulty the resin has in filling the mold cavity. Now, comparing these results with those presented in Figure 3 and Figure 5, the strong effect of this parameter on the velocity of the resin flow front can be seen clearly.

**Figure 8:**
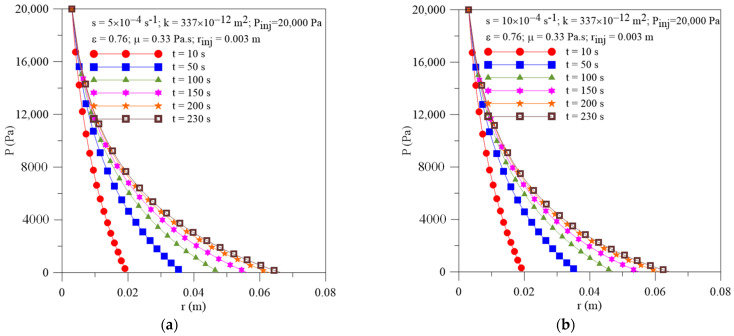
Transient behavior of pressure inside the mold for different resin sorption terms. (**a**) Case 08 and (**b**) Case 09.

**Figure 9:**
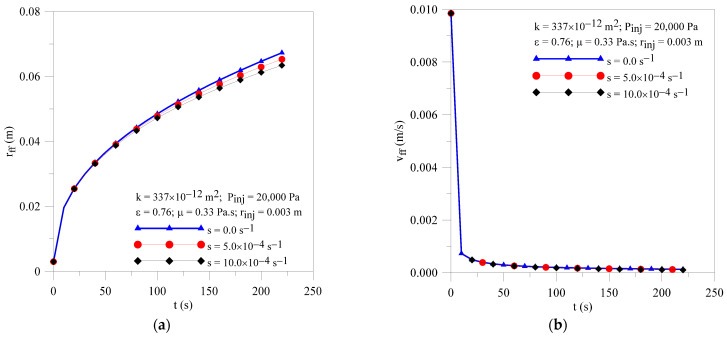
Transient behavior of (**a**) resin flow front position and (**b**) resin velocity for the Cases 02, 08 and 09.

Figure 8 shows the radial pressure field within the porous medium at different moments of the injection process and terms of resin sorption by the fibers. As expected, there are greater pressure gradients inside the mold cavity at the beginning of the injection process, which are reduced for long injection times. In addition, as can be seen, there is a small difference between the three flows’ behavior; the resin macroscopic flow front moves fast when the fibers are previously saturated or they are impermeable (s = 0.0 s ^−1^). An increase in sink term value tends to delay the resin flow inside the preform, thus increasing the final process time, as shown in Figure 9a, highlighting the role of this parameter in the progress and success of the process. Then, this phenomenon plays an important role, especially when vegetable fiber is used as reinforcement in the matrix, as this material, in general, has greater porosity when compared to synthetic fibers. Due to the small values of the resin flow front location at the beginning of the process, the effect of the resin sorption parameter on the flow front velocity is almost null, as illustrated in Figure 9b.

The fibrous preform acts as sinks, absorbing resin, thus reducing the advances of the front of resin during the infiltration process. This sink effect occurs due to the action of various process parameters such as pressure inside and outside of the fiber (free surface, capillary and vacuum pressures), fiber micropermeability, fiber geometry, fiber microporosity and fiber density, and the degree of resin saturation into the pore spaces, which has large implications in the porous media permeability estimation. The saturation degree is strongly dependent on the resin pressure surrounding the porous fiber and capillary absorption effects. In general, the capillary effects are neglected during the filling period.

**Figure 10:**
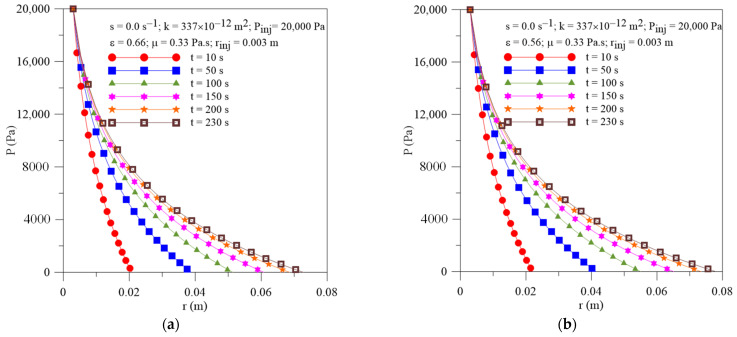
Transient behavior of pressure inside the mold for different porous media porosities. (**a**) Case 10 and (**b**) Case 11.

**Figure 11:**
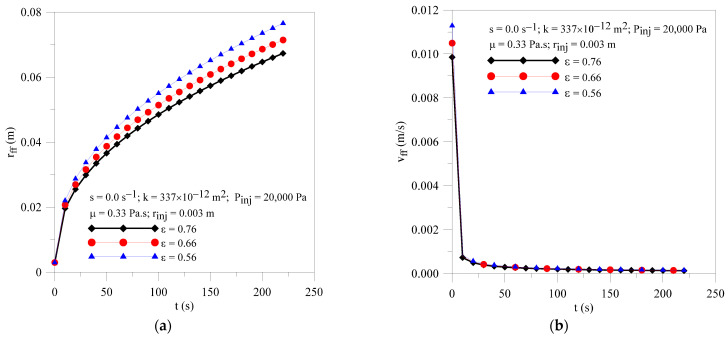
Transient behavior of (**a**) resin flow front position and (**b**) resin velocity for the Cases 02, 10 and 11.

Figure 10a,b show the radial field of pressure within the porous media at different moments of the resin infiltration process and porous media porosities (**ε** =0.56 and 0.66). At the beginning of the process, greater pressure gradients inside the mold cavity are observed for all cases studied and a decline for long process times. Furthermore, an increase in porosity tends to provoke a reduction in resin flow front location and resin velocity with the progress of impregnation time, as illustrated in Figure 11a,b, but the effect in resin velocity is strongly reduced with resin injection time.

#### 3.2.3. Effect of Resin Parameters

**Figure 12:**
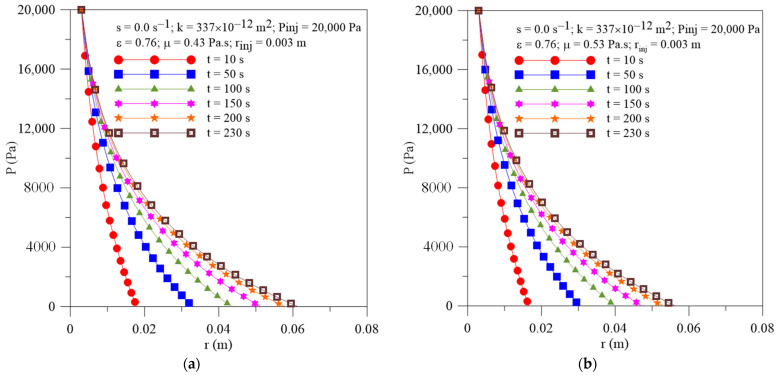
Transient behavior of pressure inside the mold for different resin viscosity. (**a**) Case 12 and (**b**) Case 13.

**Figure 13:**
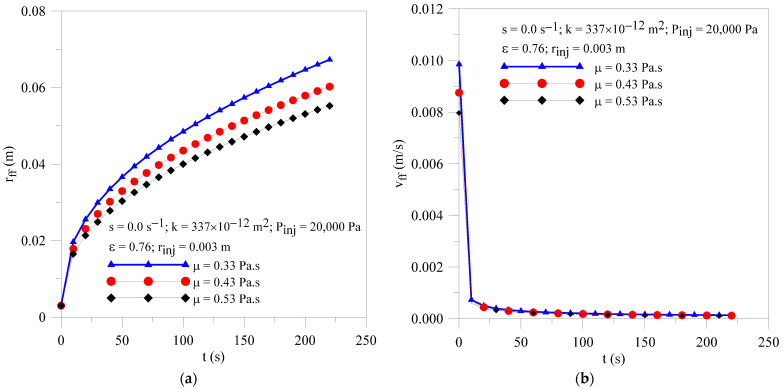
Transient behavior of (**a**) resin flow front position and (**b**) resin velocity for the Cases 2, 12 and 13.

Figure 12 shows the radial field of pressure within the porous media at different moments of the resin infiltration process and different resin viscosity. As expected, there is a transient and non-linear behavior of this parameter along the process, for all cases studied. Pressure gradients are slightly affected by increasing resin viscosity. In Figure 12a,b, the point where the curve touches the horizontal axis corresponds to the location of the resin flow front (r_ff_) at each moment of the infiltration process. Figure 13a,b illustrates the radial flow front position and resin velocity as a function of injection time, respectively. By analyzing this figure, it can be observed that an increase in resin viscosity provokes a reduction in both the velocity and radial location of the resin front at the same moment of the injection process. This phenomenon occurred due to the great mechanical friction between the resin and the mold wall, and the resin and fibers.

It is important to comment that, in accordance with Darcy´s law, the flow front velocity depends on the pressure gradient, permeability, viscosity and the distance from the inlet. Thus, the flow resistance grows with the distance from the inlet; then, the velocity drops along the flow length during the injection process for fixed inlet pressure.

In global analysis, Table 4 summarizes the flow front radial position of the fluid injected into the mold at the instant t = 230 s. By analyzing the obtained results for flow front position at the same moment of the impregnation process, it is clear that the influence of the porous media permeability, fluid viscosity and injection pressure in the fluid velocity (which is responsible for modifying the flow front radial position) is stronger than that of the sorption term and porous media porosity. The higher value of the flow front radial position (and fluid velocity) occurred in the condition of higher injection pressure, porosity and porous media permeability along with null sorption term and lower fluid viscosity. Thus, adequately controlling these process parameters becomes crucial to the success of composite manufacturing.

In practical applications, the resin viscosity decreases due to the increase in resin temperature within the mold during infiltration process; however, herein this parameter was considered to be constant. Variations in resin viscosity strongly impacts the capillary number (relationships between the viscous drag forces and surface tension forces acting across an interface between the resin and air) and formation of void spaces inside the polymer composite.

### 3.3. Weakness and Robustness of the Proposed Mathematical Model

Mathematical modeling and simulation have many benefits and disadvantages related to real physical problems. Herein, the following points on robustness and usefulness of the proposed model can be cited:

- The model can be applied to verify the effect of different process parameters, porous media and resin and to assist in understanding the process, which is potentially very important in RTM applications for composite manufacturing;
- It can be applied to validate most robust and advanced mathematical modeling, including 2D simulations, with small computational efforts;
- It can be used to quickly obtain predicted results for ideal and real physical conditions and to provide detailed information about unknown variables that cannot be directly measured by experiments in the RTM process, thus making it possible to optimize the RTM process;
- It allows for easy estimation of porous media permeability with great accuracy, an important parameter for evaluation of reinforcements;
- It includes the effect of resin sorption by fiber, which is frequently not considered in RTM simulation, highlighting the powerful force of the proposed modeling.

On the other hand, since microscopic effects were not considered in the proposed model, the following weaknesses and limitations of the modeling can be highlighted:

- Microscopic effects such as entrapped microvoids, void migration, void compression and micro flow of resin during fiber filling were not considered. Microporosity and permeability of an individual fiber are much lower than the porous media permeability and porosity. In truth, the sink term changes along the preform because it is strongly dependent on the local pressure of the resin outside the fiber.
- The viscosity of both thermoset and thermoplastic resins changes with the temperature, and here, we consider an isothermal process (no curing and crystallization processes during resin impregnation).
- In this work, effect dimension, shape and orientation of the fiber, preform deformation, fiber movement and accommodation, and fiber–fiber contact, both due to resin flow and drag forces were not considered.
- Porous preform was considered isotropic. In a true physical situation, overall permeability and porosity are affected by the fiber network, fiber packing and fiber volume fraction. In this work, variations in resin flow resistance due to actions of these parameters were neglected, and permeability, porosity and packing degree were considered to be constant and uniform.
- Variations in the mold thickness and deformations due to high pressure inside the mold cavity were neglected.

## 4. Conclusions

In this study, the RTM technique was explored on the basis of resin radial infiltration under the condition of invariant injection pressure. The porous preform filling transient process inside the closed mold cavity was modeled using the mass and momentum conservation laws coupled with the Darcy´s law and considering the effect of resin sorption by fibrous reinforcement. The research is limited to the one-dimensional approach at a macroscopic level. The method of separation of variables was used to obtain the mathematical solution of the governing equation and simulate the resin flow through the fibrous medium inserted into the mold cavity under different operating conditions. The model takes into account the effect of injection pressure, resin viscosity, injection port radius, resin sorption term, and permeability. The study was performed for a single injection gate.

From the transient results obtained in each numerical simulation, the following can be concluded: (a) the greater the resin injection pressure and the resin injection radius, the greater the location of the resin front and the velocity of the resin front will be at the same time of the process; (b) the higher the resin sorption term, the lower the position of the resin flow front and the velocity of the resin front will be, for a fixed moment of the injection process; (c) the lower the fibrous media permeability, the lower the resin front location and the resin front velocity; and (d) the lower the porous media porosity, the higher the resin flow front location and the resin front velocity. Finally, validation of the proposed modeling was confirmed by comparing the predicted transient distribution of radial pressure, resin radial front location in the mold cavity and resin filling time with results from the exact solution reported in the literature.

## Figures and Tables

**Table 1:** Mold dimensions and process parameters used in the numerical simulations.

Case	Parameter									
	Mold			Fibrous Medium			Resin	Process		
	W (m)	L (m)	h (m)	ɛ (−)	k (×10^−12^ m^2^)	s (×10^−4^ s^−1^)	μ (Pa.s)	P_inj_ (Pa)	r_inj_ (m)	P_ff_ (Pa)
01	0.15	0.32	0.0036	0.76	337.0000	0	0.33	15,000	0.003	0.0
02	0.15	0.32	0.0036	0.76	337.0000	0	0.33	20,000	0.003	0.0
03	0.15	0.32	0.0036	0.76	337.0000	0	0.33	25,000	0.003	0.0
04	0.15	0.32	0.0036	0.76	337.0000	0	0.33	20,000	0.002	0.0
05	0.15	0.32	0.0036	0.76	337.0000	0	0.33	20,000	0.001	0.0
06	0.15	0.32	0.0036	0.76	3.3700	0	0.33	20,000	0.003	0.0
07	0.15	0.32	0.0036	0.76	0.0337	0	0.33	20,000	0.003	0.0
08	0.15	0.32	0.0036	0.76	337.0000	5	0.33	20,000	0.003	0.0
09	0.15	0.32	0.0036	0.76	337.0000	10	0.33	20,000	0.003	0.0
10	0.15	0.32	0.0036	0.66	337.0000	0	0.33	20,000	0.003	0.0
11	0.15	0.32	0.0036	0.56	337.0000	0	0.33	20,000	0.003	0.0
12	0.15	0.32	0.0036	0.76	337.0000	0	0.43	20,000	0.003	0.0
13	0.15	0.32	0.0036	0.76	337.0000	0	0.53	20,000	0.003	0.0

**Table 2:** Parameters of Equation (23).

Pe (mbar)	te (s)	a_1_ (mbar s^−0.25^)	a_2_ (mbar)	a_3_ (s^−1^)	a_4_ (−)	a_5_ (s^−1^)
1888.35	281	45.20	0.18	−3099.14	−6960.16	−352.70

**Table 3:** Process parameters estimation by simulation (this work) and experiments [9].

t_border_ (s)			Parameter			
Experimental (A) [9]	Simulated (B) [This Work]	Error (%) = (A − B)/A × 100	Permeability (m^2^) [9]	s (s^−1^) [9]	Permeability (m^2^) [This Work]	s (s^−1^) [This Work]
560.00	560.50	0.09	2.00 × 10^−11^	0.0	1.48 × 10^−11^	1.0 × 10^−7^

**Table 4:** Flow front position of the fluid injected at time 230 s for all studied cases.

Case	Parameter							
	t = 230 s	Fibrous Medium			Resin	Process		
	r_ff_ (m)	ɛ (−)	k (×10^−12^ m^2^)	s (×10^−4^ s^−1^)	μ (Pa.s)	P_inj_ (Pa)	r_inj_ (m)	P_ff_ (Pa)
01	0.06078	0.76	337.0000	0	0.33	15,000	0.003	0.0
02	0.06856	0.76	337.0000	0	0.33	20,000	0.003	0.0
03	0.07532	0.76	337.0000	0	0.33	25,000	0.003	0.0
04	0.06448	0.76	337.0000	0	0.33	20,000	0.002	0.0
05	0.05881	0.76	337.0000	0	0.33	20,000	0.001	0.0
06	0.01174	0.76	3.3700	0	0.33	20,000	0.003	0.0
07	0.00406	0.76	0.0337	0	0.33	20,000	0.003	0.0
08	0.06644	0.76	337.0000	5	0.33	20,000	0.003	0.0
09	0.06444	0.76	337.0000	10	0.33	20,000	0.003	0.0
10	0.07275	0.66	337.0000	0	0.33	20,000	0.003	0.0
11	0.07798	0.56	337.0000	0	0.33	20,000	0.003	0.0
12	0.06136	0.76	337.0000	0	0.43	20,000	0.003	0.0
13	0.05625	0.76	337.0000	0	0.53	20,000	0.003	0.0

## Data Availability

The original contributions presented in this study are included in the article. Further inquiries can be directed to the corresponding author.
